# Development and validation of a live birth prediction model for expected poor ovarian response patients during IVF/ICSI

**DOI:** 10.3389/fendo.2023.1027805

**Published:** 2023-01-31

**Authors:** Xiaoyun Gong, Yunian Zhang, Yuejie Zhu, Peng Wang, Zhihui Wang, Chen Liu, Manli Zhang, Xiaolin La

**Affiliations:** ^1^ Center for Reproductive Medicine, First Affiliated Hospital of Xinjiang Medical University, Urumqi, China; ^2^ State Key Laboratory of Pathogenesis, Prevention and Treatment of High Incidence Diseases in Central Asia, Xinjiang Medical University, Urumqi, China; ^3^ Basic Medical College of Xinjiang Medical University, Urumqi, China

**Keywords:** poor ovarian response, POSEIDON criteria, IVF/ICSI transfer, nomogram model, live birth

## Abstract

**Background:**

A number of live birth predictive model during assisted reproductive technology treatment have been available in recent years, but few targeted evaluating the chances of live birth in poor ovarian response(POR) patients. The aim of this study was to develop a nomogram based on POSEIDON criteria to predict live birth in patients with expected POR.

**Methods:**

This retrospective cohort study using clinical data from 657 patients in POSEIDON Groups 3 and 4 (antral follicle count [AFC] ≤5 and AMH <1.2 ng/ml) in the Center for Reproductive Medicine, First Affiliated Hospital of Xinjiang Medical University, and Construction a nomogram model t

**Results:**

Among 657 expected POR patients, 111 (16.89%) had live births, and 546 (83.11%) did not have live births. These were divided into a training set(n=438) and a validation set (n=219). Multivariate logistic regression analysis showed that the age (OR = 0.91, 95% CI: 0.86–0.97), BMI (OR = 1.98, 95% CI: 1.09–3.67), AMH (OR = 3.48, 95% CI: 1.45–8.51), normal fertilized oocytes (OR = 1.40, 95% CI: 1.21–1.63), and the basal FSH (OR = 0.89, 95% CI: 0.80–0.98) of the female were independent factors predicting live birth in patients with expected POR. Then, an individualized nomogram prediction model was built from these five factors. The area under the ROC curve of the live birth prediction model was 0.820 in the training set and 0.879 in the validation set.

**Conclusion:**

We have developed a nomogram combining clinical and laboratory factors to predict the probability of live birth in patients with an expected POR during IVF/ICSI, which can helpful for clinician in decision-making. However, the data comes from the same center, needs a prospective multicenter study for further in-depth evaluation and validation of this prediction model.

## 1 Introduction

The rapid development of assisted reproductive technology (ART) has enabled many infertile patients to have a child by *in vitro* fertilization-embryo transfer (IVF-ET) and its expanded technologies (1). Since 1978, the world’s first test-tube baby was born, more than 6,000,000 families worldwide have achieved pregnancy through ART, bringing hope for infertile couples to conceive a child (2). In the treatment process, the controlled ovarian hyperstimulation (COH) technique can promote the simultaneous development and maturation of multiple follicles through medication, thus improving the pregnancy rate, which is crucial for IVF-ET success. However, under appropriate ovarian stimulation, the number of retrieved oocytes is still lower than expected in some patients, and this phenomenon is called poor ovarian response (POR) (3, 4). To date, the POR incidence ranges from 9% and 24% in the IVF cycle, leading to a decline of ovarian response in ART treatment, a reduction of retrieved oocytes, an increase of clinical cycle cancelation and miscarriage rate, and the risk of embryonic aneuploidy (5).

Improving the IVF-ET outcome in patients with POR has always been a focus of reproductive medicine research. The Bologna criteria developed by the European Society of Human Reproduction and Embryology (ESHRE) in 2011 are the common diagnostic criteria for POR. However, in the POR definition made by Bologna criteria, the populations with different clinical characteristics and prognosis are placed in the same category, and no further classification is made between the true positive of POR caused by low ovarian reserve and the false positive of POR caused by the deficiency of gonadotrophin (Gn) or its receptor (5).

In 2016, Alviggi et al. proposed the POSEIDON criteria as a new classification. The POSEIDON criteria divide infertile patients into four groups according to age, anti-mullerian hormone (AMH), and antral follicle count (AFC): Group 1: patients aged below 35 years with a good ovarian reserve (AFC ≥ 5, AMH ≥ 1.2 ng/mL) but unexpected POR. Group 1 is further divided into group 1a: the number of retrieved oocytes is < 4; and group 1b: the number of retrieved oocytes is 4–9 (6). Group 2: patients aged 35 years or older with good ovarian reserve (AFC ≥ 5, AMH ≥ 1.2 ng/mL) and unexpected POR. Group 2 is also further divided into group 2a: the number of retrieved oocytes is < 4; and group 2b: the number of retrieved oocytes is 4–9. Group 3: patients aged below 35 years with a diminished ovarian reserve (AFC < 5, AMH < 1.2 ng/mL). Group 4: patients aged 35 years or older with diminished ovarian reserve (AFC < 5, AMH < 1.2 ng/mL). In order to reflect the reproductive potential of these patients in a better way, the POSEIDON criteria classify patients into the “expected POR group (groups 3 and 4)” and the “unexpected POR group (groups 1 and 2)” based on POR heterogeneity (7–9). The POSEIDON criteria aim to guide individualized management and for more detailed stratification of of POR patients to reduce the population heterogeneity of the Bologna criteria

Although ART technology is slowly developing, its success rate remains approximately 40%. Its low pregnancy rate and low live birth rate enlarged both the financial and psychological burdens on infertile families and brought tremendous trouble to the clinicians (10, 11). A number of studies have predicted the pregnancy outcome after IVF/ICSI, and most of the known predictors have already become part of the conventional diagnostic procedure for infertile patients. However, few studies have predicted the pregnancy outcome for the POR patients (12, 13). Therefore, in this study, we aimed to explore the independent predictive factors associated with pregnancy outcome in POR and developed a nomogram model for live birth after IVF/ICSI procedure, in order to implement a personalized reproductive strategy for patients with expected POR.

## 2 Materials and methods

### 2.1 Subjects

This study retrospectively analyzed 3,142 patients undergoing IVF/ICSI treatment in the Reproductive Center of the First Affiliated Hospital of Xinjiang Medical University between January 2017 and December 2020 were collected. After excluding the patients with hydrosalpinx, ovarian surgery history, ovarian tumor, endometrial lesion, polycystic ovary syndrome, and the couples with chromosomal abnormalities, a total of 657 patients that conformed to POSEIDON criteria for group 3 and 4 (AFC ≤ 5, AMH < 1.2 ng/mL) were finally included. They were contained only for one cycle with transfer of one or two fresh embryos (the first cycle after fulfilling the criteria).

Our center made ovulation induction protocols according to the patients’ condition and ovarian reserve. The protocols mainly included the early-follicular-phase long-acting GnRH-agonist long protocol, Mid-luteal phase short-acting GnRH-a long protocol, antagonist protocol and natural cycle. We determine the Initial dose of gonadotropin (Merck Schlano, Italy) according to the patient`s age, BMI, AFC and previous ovarian response to stimulation. Vaginal ultrasound is used to monitor the growth of follicles every 1-2 days, and the dosage of gonadotropins is adjusted according to the patient’s peripheral blood luteinizing hormone (LH), estradiol (E2) and progesterone levels to obtain as many mature oocytes as possible. If at least 1–2 dominant follicles with a diameter of ≥ 20 mm were observed, or three follicles with a diameter of ≥ 18 mm had been monitored previously, the blood E2 level would be measured to determine whether to stop the Gn injection, and determine the recombinant human chorionic gonadotropin (hCG) injection dosage. When the leading follicles reached a mean diameter of 14 mm, 0.25mg hCG (Livzon Pharmaceuticals, China) was given to trigger ovulation. Transvaginal ultrasound-guided oocyte retrieval was performed at 34–36 hours after HCG injection. Embryos were transferred on day 3. Progesterone was injected vaginally or intramuscularly daily during the luteal phase until 2 weeks after ET.

### 2.2 Data collection

The baseline data and clinical characteristics of patients were collected to analyze the POR predictors. The categorical variables included the education background, infertility type, and pregnancy history. The continuous variables included age, body mass index (BMI), age of menarche, average menstrual cycle, and duration of infertility; AFC on the third day of the menstrual cycle, endometrial thickness, LH, E2, progesterone, FSH, and AMH levels; dosing days, initial dose and total dose of Gn, endometrial thickness and hormone levels on the day of HCG injection. BMI greater than 24 kg/m^2^ can be diagnosed as overweight. AFC was detected using transvaginal ultrasonography on the third day of the menstrual cycle. Endometrial thickness was observed on the day of HCG injection, and endometrial thickness >7 mm is endometrial receptivity markers as prognostic factors for conceiving.

Normal fertilization was confirmed when the presence of two pronuclei (2PN) and the extrusion of the second polar body(2PB) were observed 16–18 h after IVF/ICSI (14, 15). Cleavage embryos were classified as high-quality embryos (grade I and II embryos) if they had seven to nine cells on day 3 and as same-sized blastomeres if with less than 20% blastomeric fragments. Embryos graded III or IV including those that had less than seven cells on day 3, and no less than 20% fragmentation were called poor quality.

### 2.3 Primary outcome

The primary outcome of the nomogram was live birth, defined as a pregnancy that continues with at least one live-born fetus after 28 weeks of gestation and survived for more than one month.

### 2.4 Data analysis

All data were performed using R software (version 4.0.2). Patients were divided into a training set and a validation set by the sampling techniques of random numbers.The bilateral p-values < 0.05 were considered statistically significant. The mean± standard deviation (SD) of continuous variables were compared using the t-test, analysis of variance, or Mann–Whitney U test, and the numbers (percentages) of categorical variables were compared using the χ^2^ test or Fisher’s exact test.The univariate and multivariate logistic regression analyses were performed on the influencing factors. The statistically significant variables were included in the multivariate logistic regression analysis, finally selected into the model *via* the forward stepwise approach, and visualized as the nomogram scoring system. The nomogram scoring system was evaluated as follows: (1) the discrimination power of the nomogram scoring system was evaluated by the area under the ROC curve; (2) the prediction accuracy was evaluated by the calibration curve; (3) the clinical practicality of the nomogram scoring system was evaluated.

### 2.5 Ethical statement

This study was approved by the Ethics Committee of the First Affiliated Hospital of Xinjiang Medical University. In this study, all enrolled patients provided their formal consent. The data of this study were analyzed anonymously and the personal identifiers were completely removed. This study was conducted in compliance with the “Declaration of Helsinki”.

## 3 Results

### 3.1 Analysis of patients’ baseline information

Between January 2017 and December 2020, 657 patients meeting the POSEIDON criteria for the “expected POR group” were included in this study. These patients were allocated into either training set (n = 438) or validation set (n = 219) for model establishment and validation. The baseline characteristics were shown in **Table 1**, and no significant difference was noted between the two groups (P > 0.05). In the training set, live birth was achieved in 80 patients (15.98%).

**Table 1:** Baseline characteristics of study population.

Characteristics	Training set (n = 438)	Validation set (n = 219)	P value
Age (years)	36.77 (23–49)	36.00 (26–48)	0.083
BMI (kg/m^2^)	23.33 ± 3.37	22.83 ± 2.99	0.061
Education background			0.552
Primary school	12 (2.74%)	8 (3.65%)	
Middle school	99 (22.60%)	57 (26.03%)	
Bachelor degree	299 (68.26%)	144 (65.75)	
Graduate degree or above	28 (6.39%)	10 (4.57%)	
Type of infertility			0.912
Primary infertility	194 (44.29%)	98 (44.75%)	
Secondary infertility	244 (55.71%)	121 (55.25%)	
Duration of infertility (years)	4.05 ± 3.01	4.55 ± 3.28	0.087
AFC	2.88 ± 1.31	2.80 ± 1.43	0.676
AMH (ng/mL)	0.50 ± 0.35	0.53 ± 0.28	0.059
Basal FSH (IU/L)	7.60 ± 3.62	7.69 ± 3.60	0.738
Basal LH (IU/L)	4.42 ± 2.23	4.51 ± 1.75	0.056
Basal E2 (ng/L)	184.91 ± 74.73	179.54 ± 73.77	0.412
Basal P (μg/L)	0.84 ± 0.43	0.86 ± 0.38	0.148
FSH on HCG day (IU/L)	6.02 ± 1.72	6.16 ± 1.95	0.192
LH on HCG day (IU/L)	4.05 ± 1.36	4.05 ± 1.54	0.297
Endometrial thickness on HCG day	10.50 ± 2.07	10.83 ± 2.10	0.069
Retrieved oocytes	5.06 ± 3.45	5.34 ± 4.74	0.341
normal fertilized oocytes	3.34 ± 1.96	3.33 ± 2.82	0.088
Quality of the transferred embryos			0.603
One or two high-quality embryos	287(65.53%)	139(63.47%)	
One high-quality embryo and one poor-quality embryo	151(34.47%)	80(36.53%)	
Initial dose of Gn	209 ± 89.247	216.78 ± 80.81	0.742
Total dose of Gn	2647.36 ± 597.41	2633.35 ± 541.36	0.796
Dosing days of Gn	12.27 ± 2.53	12.50 ± 2.18	0.261
pregnancy outcome			0.377
pregnancy	80 (18.26%)	31 (18.72%)	
unpregnancy	358 (81.74%)	188 (81.28%)	

### 3.2 Logistic regression analysis of live birth

The univariate and multivariate logistic regression analyses of live birth in the training set is shown in **Table 2**. According to the univariate analysis, the live birth in expected POR patients was associated with their age, BMI, AMH, normal fertilized oocytes, basal FSH, and total Gn (P < 0.05). Statistically significant variables were screened out by the univariate analysis and entered into the unconditional binary multivariate logistic regression. Multivariate logistic regression analysis showed that female age (OR = 0.91, 95% CI: 0.86–0.97), BMI (OR = 1.98, 95% CI: 1.09–3.67), AMH (OR = 3.48, 95% CI: 1.45–8.51), normal fertilized oocytes (OR = 1.40, 95% CI: 1.21–1.63), and the basal FSH (OR = 0.89, 95% CI: 0.80–0.98) were significantly associated with live birth in expected POR patients. Collinearity diagnostics to the above independent factors were performed. The variance inflation factors (VIFs) were 1.142, 1.007, 1.193, 1.298, and 1.009, respectively, suggesting no multicollinearity among the five independent factors.

**Table 2:** Univariate and multivariate logistic regression models in the training set.

	Univeriate analysis		Multivariate analysis	
	OR (95% CI)	P value	OR (95% CI)	P value
age	0.93 (0.86–0.99)	0.04	0.92 (0.87–0.97)	0.005
Infertility year	0.99 (0.88–1.10)	0.85	NA	
Secondar infertility	0.73 (0.36–1.44)	0.36	NA	
BMI ≤ 24	2.09 (1.11–4.03)	0.02	1.92 (1.07–3.51)	0.030
AMH	2.65 (1.02–7.01)	0.04	2.64 (1.09–6.44)	0.031
Oocytes retrieved	1.28 (1.09–1.50)	0.00	1.31 (1.18–1.47)	<0.001
Transferred One high-quality embryo and one poor-quality embryo	0.914(0.65-1.28)	0.60	NA	
AFC	1.09 (0.85–1.39)	0.50	NA	
normal fertilized oocytes	0.99 (0.83–1.19)	0.96	NA	
education background middle school	0.24 (0.03–2.21)	0.16	NA	
education background bachelor degree	0.68 (0.12–5.60)	0.68	NA	
education background Graduate degree or above	0.68 (0.09–7.12)	0.72	NA	
Basal FSH (IU/L)	0.89 (0.79–0.98)	0.02	0.89 (0.80–0.98)	0.023
Basal LH (IU/L)	1.02 (0.88–1.17)	0.75	NA	
Basal E2 (ng/L)	0.99 (0.99–1.00)	0.17	NA	
Basal P (μg/L)	1.62 (0.80–3.27)	0.18	NA	
FSH on HCG day (IU/L)	0.86 (0.71–1.03)	0.11	NA	
LH on HCG day (IU/L)	0.99 (0.72–1.32)	0.96	NA	
E2 on HCG day (IU/L)	1.00 (0.99–1.00)	0.21	NA	
P on HCG day (IU/L)	0.99 (0.86–1.16)	0.96	NA	
endometrial_thickness>7mm	0.78 (0.40–1.53)	0.46	NA	
Initial dose of Gn	1.00 (0.99–1.00)	0.22	NA	
Dosing days of Gn	1.14 (0.96–1.36)	0.14	NA	
Total dose of Gn	1.00 (1.00–1.01)	0.84	NA	

### 3.3 Development and validation of the nomogram

**Figure 1:**
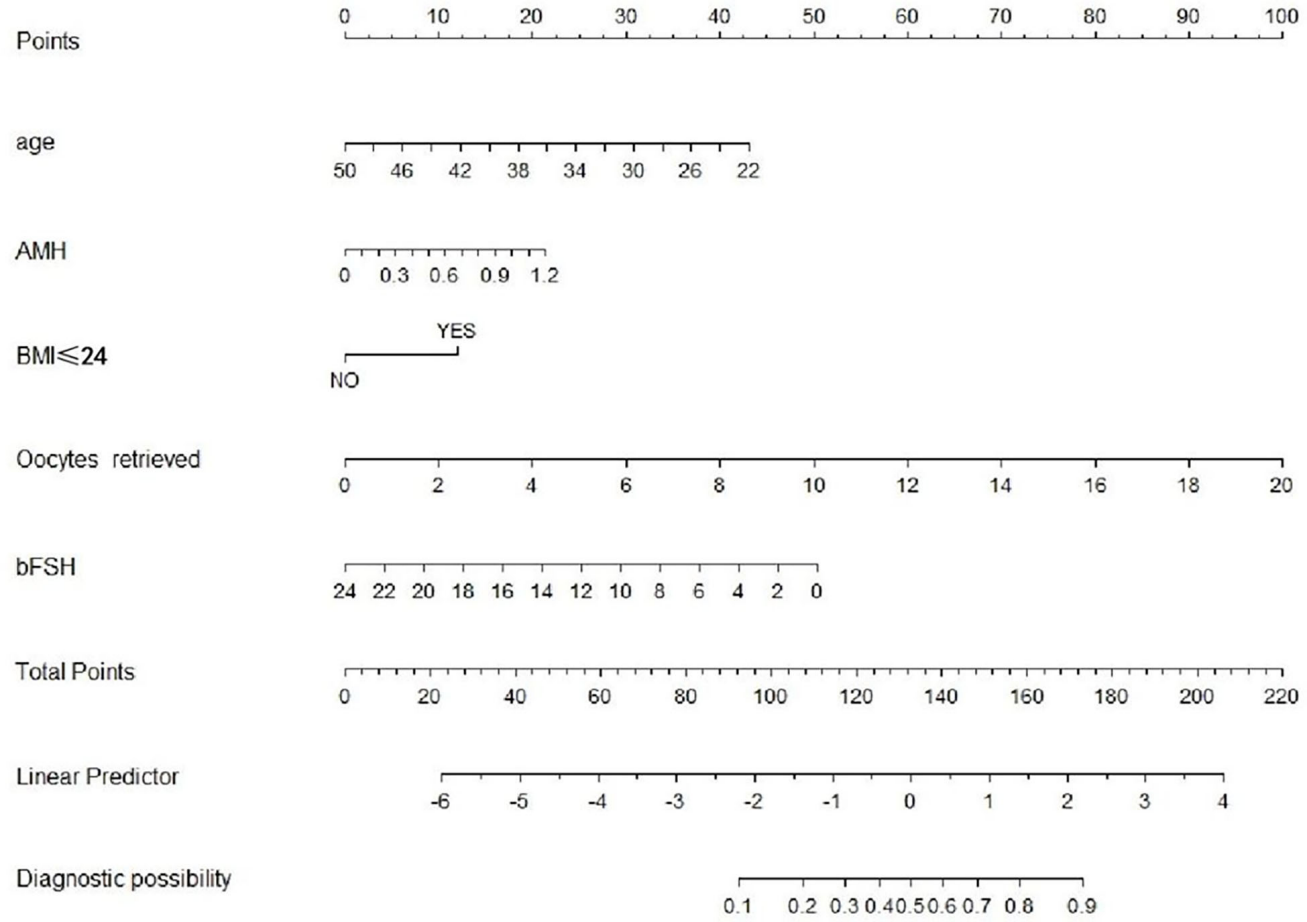
Nomogram for predicting the probability of live birth in patients with expected POR.

We performed five independent factors in the prediction model. Next, the training set was used to build the individualized nomogram prediction model. According to the nomogram, the corresponding score of each predictor was obtained. The probability of live birth in patients with expected POR can be derived from the sum of these scores, as shown in **Figure 1**.

**Figure 2:**
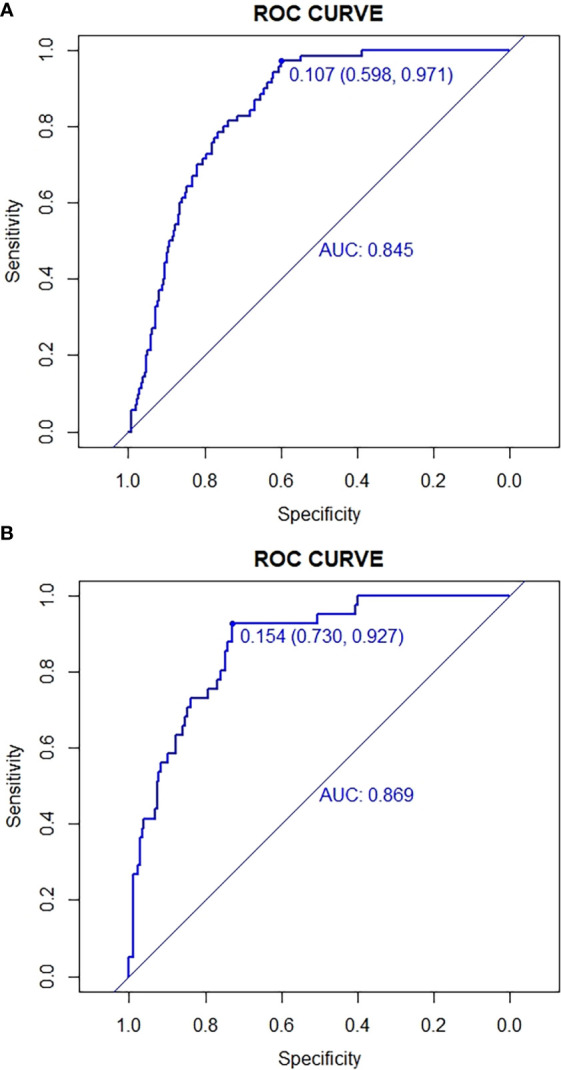
ROC curves for validating the discrimination power of the nomogram. **(A)** Development group. **(B)** Validation group.

**Figure 3:**
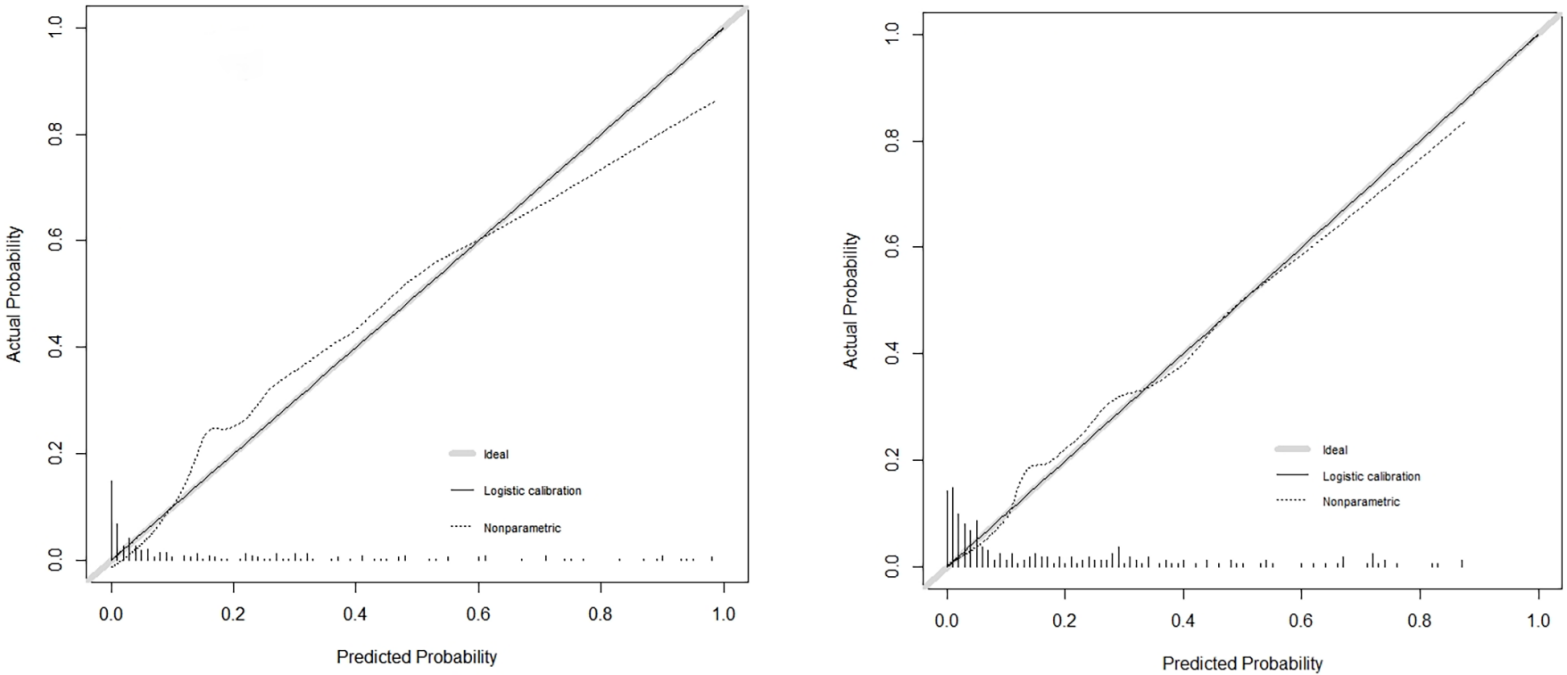
Calibration plots of the nomogram for the probability of the development group and validation group.

The predictive effect of the nomogram scoring system was evaluated using the ROC curve and its area under the curve (AUC). The area under the ROC curve of the live birth prediction model for patients with POR was 0.820 in the training set (**Figure 2**) and 0.879 in the validation set (**Figure 3**). This value indicated that the nomogram model has a high-quality predictive value.

## 4 Discussion

Some clinical prediction models have been developed to estimate the chances of IVF success in different populations over the last three decades (16, 17). In a clinical pregnancy failure prediction model based on 281 POR patients, is still conducted according to the Bologna criteria, cannot be distinguished the biological heterogeneity of patients with POR (18, 19). Another study found independent predictors of live birth in POR patients based on the POSEIDON criteria, but failed to develop a complete live birth predictive model (20). Conforti et al. found that, with the increasing age of the first delivery in recent years, the proportion of patients in group 4 under POSEIDON criteria is as high as 55%, and the proportion of patients in group 3 is about 10% (21). Diminished ovarian reserve becomes a common feature of these two cohorts. However, the factors contributing to this “expected” POR remain uncertain and it has been difficult to develop clinical management strategies based on the characteristics and prognoses of POR patients. Therefore, our study has developed a complete nomogram with external validations for expected POR, and the performance is quite good (AUC of 0.820). We hope that this nomogram will provide useful counseling tools regarding pregnancy prospects for POR patients and guide clinicians in their assessment of IVF-ET treatment outcomes.

Consistent with multiple studies, we uncovered that age is a critical factor influencing the ART outcome in POR patients (22). A classic report on the impact of a woman’s age on fertility indicated that, as the age increasing, the female infertility rate also escalates: 6% in women aged 20–24 years, 9% in women aged 25–29 years, 15% in women aged 30–34 years, 30% in women aged 35–39 years, and 64% in women aged 40–44 years (23). The ovarian reserve of female gradually declines with the increasing age, and the quantity and quality of oocytes decrease significantly, which severely negatively impacts the successful pregnancy of patients undergoing IVF/ICSI treatment (24). The large-cohort retrospective study conducted by Hogan et al. found that oocyte donor age is a key factor affecting the oocyte recipient’s cumulative live birth rate, but the oocyte recipient age is irrelative with the cumulative live birth rate. Additionally, the pregnancy rate is significantly higher in recipients of oocytes from oocyte donors aged below 35 years than in recipients of oocytes from oocyte donors aged above 35 years (25). In this study, the nomogram obtained from the univariate and multivariate logistic regression analyses revealed that the clinical pregnancy rate of expected POR patients decreased with age, which is consistent with the observations from previous studies.

Obesity will affect female fertility. A study has shown that the elevated insulin levels in obese patients will stimulate the production of ovarian androgens, which can be converted into more estrogens, formulating a negative feedback on the hypothalamic-pituitary-ovarian axis, thus suppress FSH formation, which may exhibit as the ovulatory dysfunction caused by the growth of non-dominant follicles (26). There are different opinions among the researchers towards the impact of BMI on ovulation induction in IVF. Sarais et al. categorized 1602 fresh embryo transfer recipients according to BMI, and concluded that there is no significant difference among the groups regarding to the total dose of Gn for ovarian stimulation, the duration of Gn effect, the number of oocytes retrieved, and the fertilization (27). While our study indicated that a high total dose of Gn is required for obese individuals, but no difference is observed among various BMI groups in the initial dose of Gn for ovarian stimulation and duration, which is consistent with the meta-analysis by Sermondade et al. (28). Obesity may affect the ovarian response to Gn or drug metabolism, and lower the *in vivo* effective concentration of drugs. Therefore, reducing the body weight prior to an IVF cycle may improve the outcome of IVF cycle in over-weighted women.

AMH can inhibit the proliferation and growth of follicles by limiting the functions of growth factors and Gn. It reflects primordial follicles’ condition directly and predicts the patient’s ovarian reserve effectively. Also, its stability is not affected by the menstrual cycle (29). Morin et al. demonstrated that more than 0.09 embryos would be obtained for every additional 0.1 ng/mL AMH; patients with AMH value higher than 0.52 ng/mL would obtain at least 0.62 more embryos than those with AMH value less than 0.25 ng/mL (30). Our previous research demonstrated that AMH had a linear and specific quantitative correlation with the number of embryos per stimulation cycle in POR patients. Similarly, AMH could evaluate the number of embryos effectively in patients with expected POR (31). Again, our conclusions that AMH is strongly associated with the pregnancy outcome in patients with expected POR, which is in line with the findings of other studies in the field (32).

Estrogen levels in females would be reduced as the decline of ovarian reserve, resulting in an increase of FSH secretion. The elevated basal FSH (bFSH) level is associated with the declined ovarian reserve (33). Therefore, bFSH has been widely used in reproductive medicine to evaluate the ovarian reserve and its response. Some studies suggested that the serum level of bFSH will increase significantly only when the ovarian reserve is severely declined, and it fluctuates greatly during and within the menstrual cycles, therefore bFSH has no optimal sensitivity or specificity on ovarian response prediction (34). In this study, multiple indicators were applied to evaluate the pregnancy outcome in POR patients, which could effectively improve the prediction accuracy.

The number of retrieved oocytes is quite essential for the outcome of ART treatment. Retrieved oocytes can only be fertilized at metaphase II (MII). Limited retrieved oocytes result in less excellent embryos or even no embryo available for transfer, which eventually ends up with a high IVF cycle cancelation rate and a major reduction in the clinical pregnancy rate. A study of 1706 IVF cycles in a conventional long-term protocol showed that, for the young patients with less than 5 oocytes retrieved, the clinical pregnancy rate is only 14% and the IVF cycle cancelation rate is up to 40% (35). Figueira Rde et al. concluded that, there is no significant difference in fertilization rate, excellent embryo rate, and implantation rate between patients aged below 35 years with four or less MII oocytes and patients with a normal ovarian response; they also observed that the small number of retrieved oocytes largely affected embryo quantity rather than embryo quality in young patients (36). Therefore, our study showed that the number of retrieved oocytes is an effective indicator for pregnancy outcome prediction in patients with expected POR. As more oocytes are retrieved, there will be a higher chance to obtain more fertilized oocytes and excellent embryos.

Validation of the nomogram is crucial to avoid the model overfitting and determine its generalizability. In this study, the calibration curve demonstrated the best concordance between the prediction and actual observation, thus guaranteed the reproducibility and reliability of the developed nomogram. This user-friendly scoring system enables both the clinicians and the patients to make individual prognosis of the pregnancy outcome. Nevertheless, our study still has shortcomings: (1) This is a retrospective study, and selection bias cannot be fully avoided. However, strict inclusion criteria were developed based on the POSEIDON criteria, and sufficient clinical samples were collected so that the study population could truly reflect the situation of the disease occurrence. (2) The data for this study were obtained from the same center. Although patient samples from different periods were used to validate the model, we still need evidence from other centers for validation. Therefore, we would perform a prospective multicenter study for further in-depth evaluation and validation of this prediction model.

## Data availability statement

The original contributions presented in the study are included in the article/supplementary material. Further inquiries can be directed to the corresponding author.

## Ethics statement

The studies involving human participants were reviewed and approved by This study was approved by the Ethics Committee of the First Affiliated Hospital of Xinjiang Medical University. Written informed consent for participation was not required for this study in accordance with the national legislation and the institutional requirements.

## Author contributions

GX and YNZ: Conceived the study and manuscript writing. YJZ and PW: Data acquisition. ZW: Editing of this manuscript. CL: Clinical consultation. MZ: Data preprocessing. XL: Resources, study design, supervision, and finally manuscript approval. All authors contributed to the article and approved the submitted version.
